# Sarcopenia and Appendicular Muscle Mass as Predictors of Impaired Fasting Glucose/Type 2 Diabetes in Elderly Women

**DOI:** 10.3390/nu13061909

**Published:** 2021-06-02

**Authors:** Carola Buscemi, Yvelise Ferro, Roberta Pujia, Elisa Mazza, Giada Boragina, Angela Sciacqua, Salvatore Piro, Arturo Pujia, Giorgio Sesti, Silvio Buscemi, Tiziana Montalcini

**Affiliations:** 1 Department of Clinical and Molecular Medicine, Postgraduate Specialization School in Geriatrics, University of Catania, 95100 Catania, Italy; carola.buscemi@gmail.com (C.B.); salvatore.piro@unict.it (S.P.); 2 Unit of Clinical Nutrition, Dipartimento di Promozione della Salute, Materno-Infantile, Medicina Interna e Specialistica di Eccellenza (PROMISE), University of Palermo, AOU Policlinico “P. Giaccone”, 90127 Palermo, Italy; silvio.buscemi@unipa.it; 3 Department of Medical and Surgical Science, University Magna Graecia, 88100 Catanzaro, Italy; yferro@unicz.it (Y.F.); robertapuj@gmail.com (R.P.); elisamazza@inwind.it (E.M.); giadyboragina@gmail.com (G.B.); sciacqua@unicz.it (A.S.); pujia@unicz.it (A.P.); 4 Dipartimento di Medicina clinica e molecolare, Università la Sapienza, 00185 Roma, Italy; giorgio.sesti@uniroma1.it; 5 Department of Clinical and Experimental Medicine, University Magna Grecia, 88100 Catanzaro, Italy

**Keywords:** diabetes, aging, nutrition, body composition, sarcopenia, appendicular skeletal muscle mass

## Abstract

Elderly women exhibit a high risk of type 2 diabetes (T2D), but no definitive data exist about the possible role of postmenopausal increases in visceral adiposity, the loss of lean body mass, or decreases in the sum of the lean mass of arms and legs (appendicular skeletal muscle mass (ASMM)). This retrospective, longitudinal study investigated whether body composition (bioelectrical impedance analysis) predicted the development of impaired fasting glucose (IFG) or T2D in a cohort of 159 elderly women (age: 71 ± 5 years, follow-up: 94 months) from southern Italy (Clinical Nutrition and Geriatric Units of the “Mater Domini” University Hospital in Catanzaro, Calabria region, and the “P. Giaccone ”University Hospital in Palermo, Sicily region). Sarcopenia was defined in a subgroup of 128 women according to the EWGSOP criteria as the presence of low muscle strength (handgrip strength <16 kg) plus low muscle mass (reported as appendicular skeletal muscle mass <15 kg). Participants with a low ASMM had a higher IFG/T2D incidence than those with a normal ASMM (17% vs. 6%, p-adjusted = 0.044); this finding was independent of BMI, fat mass, waist circumference, and habitual fat intake (OR = 3.81, *p* = 0.034). A higher incidence of IFG/T2D was observed in the subgroup with sarcopenia than those without sarcopenia (33% vs. 7%, p-adjusted = 0.005) independent of BMI and fat mass (OR = 6.75, *p* = 0.007). In conclusion, this study demonstrates that elderly women with low ASMM had a higher probability of developing IFG/T2D. Further studies are needed to confirm these results in men and in other age groups.

## 1. Introduction

Despite significant advances in diagnosis, monitoring, and treatments, type 2 diabetes (T2D) and its complications remain among the major causes of morbidity and mortality [1,2,3]. The aging of the population is a significant driver of the diabetes epidemic. The rate of T2D is considerably increased in older adults, with a prevalence ranging from 25% to 35% [4]. Older adults with T2D also have the highest rates of complications [5,6], including cardiovascular events, peripheral neuropathy, and disability [7,8]. Furthermore, it is widely recognized that the association between T2D and the onset of its complications differs significantly according to sex; indeed, this association is stronger in diabetic women than in men [9,10,11,12,13,14,15,16,17,18]. In particular, diabetic women could have a marked clustering of cardiovascular risk factors [9,10,11,12,13,14,15,16,17,18].

A biological reason for the increased risk of T2D and its complications in elderly women might be the propensity for insulin resistance (IR) that is driven by increased visceral adiposity in the postmenopausal period [15]. Adiponectin, which is inversely correlated with estradiol in postmenopausal women, seems to be the most interesting molecule released from fat cells with profound protective actions in the pathogenesis of diabetes mellitus [19]. It has been demonstrated that a decreased adiponectin level caused by obesity-induced IR in the adipose tissue leads to decreased insulin sensitivity in the liver and skeletal muscle and, consequently, to IR-related metabolic phenotypes [19]. At the same time, after menopause, adipose tissue represents the primary source of estrogen production in the body through the aromatization of androgens [20]. Aside from the belief that estrogens impair carbohydrate metabolism, circulating estrogens in postmenopausal women are only viewed as biomarkers of the expanded adipose tissue mass and represent biomarkers for the pathological process that predisposes them to T2D [20,21]. However, overall, prospective studies that measured fat mass (FM) [16,17,22] or lean body mass (LBM) [23,24,25] raised doubts about the prevailing role of adiposity in the pathophysiology of T2D. Although it was demonstrated that individuals with diabetes have both higher FM and lower LBM than nondiabetic people [26], the combination of high FM and high LBM is also associated with an elevated risk of developing T2DM [27]. Interestingly, it was observed that elderly individuals with T2D had both low appendicular LBM and low muscle quality [28], which are two characteristic components of sarcopenia in the elderly [29]. Older people have less appendicular skeletal muscle mass (ASMM) than younger individuals [30]. However, studies assessing the predictive capacity of ASMM to identify the incidence of T2D are lacking.

Therefore, the aim of our study was to investigate whether, in addition to obesity and body composition (total fat mass and fat-free mass), ASMM is able to predict the development of prediabetes (impaired fasting glucose) or diabetes in elderly women.

## 2. Materials and Methods

**Figure 1:**
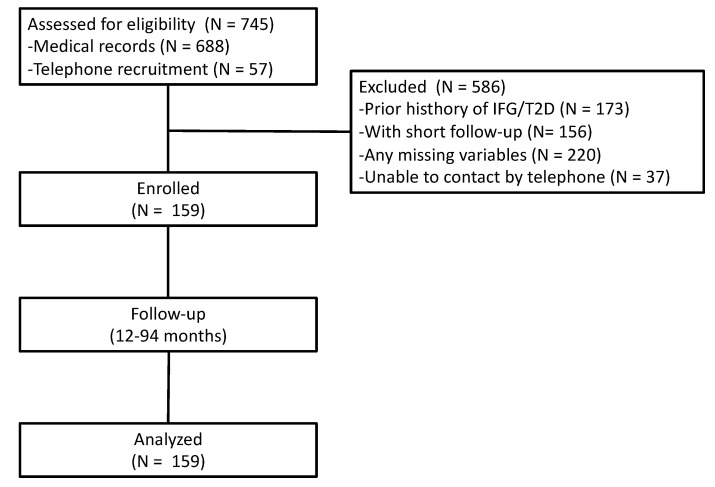
Flowchart of the participants in the study. IFG, impaired fasting glucose; T2D, type 2 diabetes.

In this multicenter, retrospective cohort study, we selected a population of 745 elderly women (≥65 years) who were consecutive outpatients undergoing health screening tests at the Clinical Nutrition and Geriatric Units of the “Mater Domini” University Hospital in Catanzaro (Italy) and the “P. Giaccone” University Hospital in Palermo (Italy) between October 2012 and April 2021; these patients had at least one follow-up visit. Data that were available from existing databases were obtained for research purposes. Individuals were excluded if they had type 1 or 2 diabetes or had followed a special diet and/or used any dietary supplements in the three months prior to the follow-up visit. All patients included in the study did not suffer from any chronic diseases, such as CKD-EPI stage 2–5 chronic kidney disease, liver cirrhosis, chronic obstructive pulmonary disease, thyroid dysfunction, heart failure ≥NYHA class 2, or any malignant cancer diagnosed in the last 5 years. Additionally, the included patients were not taking anti-obesity, antidiabetics, or psychotropic drugs, according to their medical history, physical examinations, and laboratory tests. Baseline and follow-up clinical characteristics of the population, as well as biochemical parameters, were obtained from their clinical records. Among those initially included, 159 patients completed a full baseline nutritional and biochemical assessment and met all eligibility criteria. Of these patients, 57 completed the enrollment by telephone to confirm their inclusion criteria eligibility (Figure 1).

The study was conducted in accordance with the Declaration of Helsinki and the protocol was approved by the Ethics Committee of the “Mater Domini” University Hospital of Catanzaro, Italy (project identification code no. 23, 21 January 2021) and by the Ethics Committee “Palermo 1” of the Policlinico “P. Giaccone” University Hospital (project identification code no. 3/2015, 11 March 2015). All subjects gave their written informed consent for inclusion before they participated in the study. The data obtained from the cohort of the Palermo center were part of The Nutrition, Cardiovascular Wellness and Diabetes (ABCD_2) project (ISRCTN15840340). This study was a longitudinal observational single-center study of a cohort that was representative of the general population living in Palermo, which is the largest city in Sicily (Italy).

### 2.1. Diabetes, Impaired Fasting Glucose, and Cardiovascular Risk Factor Assessment

Glucose tolerance was classified according to fasting blood glucose concentrations. In particular, diabetes was diagnosed if the fasting blood glucose concentration was ≥126 mg/dL (7 mmol/L) or antidiabetic treatment was administered; impaired fasting glucose (IFG), a condition of prediabetes, was diagnosed if the fasting blood glucose concentration was in the range of 100–125 mg/dL (5.5–6.9 mmol/L) [31]. Hyperlipidemia was defined as blood concentrations of total cholesterol >200 mg/dL and/or triglycerides >200 mg/dL or the use of lipid-lowering drugs. Hypertension was diagnosed if systolic blood pressure (SBP) was ≥130 mmHg and/or diastolic blood pressure (DBP) ≥85 mmHg or if antihypertensive medications were used. Current smokers who smoked >100 cigarettes in their lifetime and smoke cigarettes every day or some days were considered [32,33].

### 2.2. Anthropometric and Dietary Intake Assessments

Body weight (BW) and waist and hip circumferences (WC and HC) were obtained from medical records. Body mass index (BMI) was calculated as weight (kg)/height (m)^2^. Obesity was diagnosed if the BMI was ≥30 kg/m^2^.

Hand-to-foot bioelectrical impedance analysis (BIA) (BIA-EFG electrofluid graph, Akern srl, Florence, Italy) was performed to estimate the percentage of FM [34], phase angle (pA), and ASMM according to the manufacturer’s equations (Akern, Bodygram Plus software) [14,35]. We considered the following cutoff value for the definition of obesity in women: FM ≥ 35% [36,37]. The ASMM represents the sum of the muscle mass of the arms and legs. According to the European Working Group on Sarcopenia in Older People (EWGSOP), we used a cutoff value for women of 15 kg for diagnosing BIA-derived low ASMM [29].

Sarcopenia was defined as a syndrome that was characterized by progressive and generalized loss of skeletal muscle mass and strength with a risk of adverse outcomes [29]. We used the EWGSOP criteria, which include the presence of low muscle strength (handgrip strength (HGS) <16 kg was defined as low muscle strength) plus low muscle mass reported as ASMM (<15 kg was defined as low ASMM). HGS was measured as previously described [38] using a hydraulic hand dynamometer (Hersteller/manufacturers, SAEHAN Corporation, Masan-Korea).

Dietary intake was assessed via a combination of a validated food frequency questionnaire and a 7-day food record, and it was calculated using the MetaDieta 3.0.1 nutritional software (Metedasrl, San Benedetto del Tronto, Italy) [38,39]. The nutrient database used to calculate the nutrient intake was primarily derived from INRAN (National Institute of Food Research) 2000 and IEO (European Institute of Oncology) 2008.

### 2.3. Biochemical Evaluation

All laboratory parameters were obtained from medical records. Data relating to blood concentrations of glucose, total cholesterol, high-density lipoprotein (HDL) cholesterol, triglycerides, and creatinine were recorded. Low-density lipoprotein (LDL) cholesterol levels were calculated using Friedewald’s equation [40].

### 2.4. Statistical Analysis

Data are reported as the means ± standard deviations (SD). For the purposes of this study, a minimum sample size of 150 individuals (Z-statistic) was calculated to find a 10% difference in the incidence of T2D between normal- and low-ASMM groups by considering a cumulative incidence of diabetes in European older adults ranging from 5% to 8% over a 6-year follow-up [41] with 80% power on a two-sided level of significance.

According to the EWGSOP criteria [29,38], we categorized the enrolled population into the following groups: (1) normal ASMM, (2) low ASMM, (3) with sarcopenia, and (4) without sarcopenia. Between-group differences were compared using Student’s unpaired *t*-test. A Kaplan–Meier estimation of survival curves with Mantel–Cox log-rank univariate analysis was performed to identify any differences in the cumulative risk of developing IFG/T2D between the normal ASMM and low ASMM groups. A multivariate Cox proportional hazard model was used to adjust for potential confounding factors, which were the variables that significantly differed according to the *t*-test. Categorical variables were analyzed using a Mantel–Haenszel chi-square test to assess the odds ratios. The categorizations of normal ASMM and low ASMM, as well as normal and sarcopenic individuals, were used to construct a two-by-two contingency table. A general linear model (GLM) was used to adjust the prevalence of confounding factors (such as BMI, FM, CV, and dietary fats at baseline). A chi-square test was also used to compare the incidence between the following groups: normal ASMM/high FM, normal ASMM/normal FM, low ASMM/high FM, and low ASMM/normal FM. We then performed a ROC curve to identify the best cut-off of ASMM to detect the onset of IFG/T2D.

All analyses were performed using SPSS 25.0 software for Windows (S. Wacker Drive, Chicago, IL, USA). A two-tailed *p*-value of <0.05 was considered to be statistically significant.

## 3. Results

The demographic and clinical characteristics of the participants are presented in Table 1.

The mean age was 71 ± 5 years. The prevalences of obesity at the baseline were 26% and 38% according to the BMI- and BIA-derived FM% categories, respectively. The prevalence of sarcopenia at the baseline was 9.4% and low ASMM was 45% (Table 1). The characteristics of the population categorized as normal and low ASMM are presented in Table 2.

The Cox hazard analysis demonstrated that only low ASMM increased the risk for IFG/T2D (HR 5.30, 95% CI: 1.34–21.04, *p* < 0.017); BMI, high FM, WC, and the habitual intake of dietary fats were not associated with IFG/T2D onset (Table 3).

**Figure 2:**
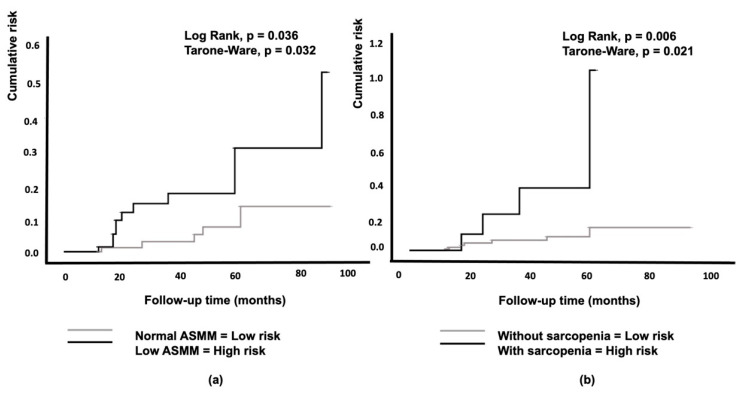
Kaplan–Meier curves for the cumulative risk of impaired fasting glucose/type 2 diabetes according to appendicular skeletal muscle mass (**a**) and the diagnosis of sarcopenia (**b**). ASMM, appendicular skeletal muscle mass.

The Kaplan–Meier curves showed that low ASMM was associated with a significantly higher probability of developing IFG/T2D over a period of 94 months compared to normal ASMM conditions (Figure 2a).

Cox hazard analysis of sarcopenia (ASMM-HGS diagnosed, *n* = 128 individuals), BMI, and high FM indicated that only sarcopenia was significantly associated with the risk of IFG/T2D (HR 4.72, CI 1.38–16.18, *p* < 0.013) (Table 3, Figure 2b). Among the obese individuals, the 7-year incidence of diabetes was 8% (data not shown).

**Figure 3:**
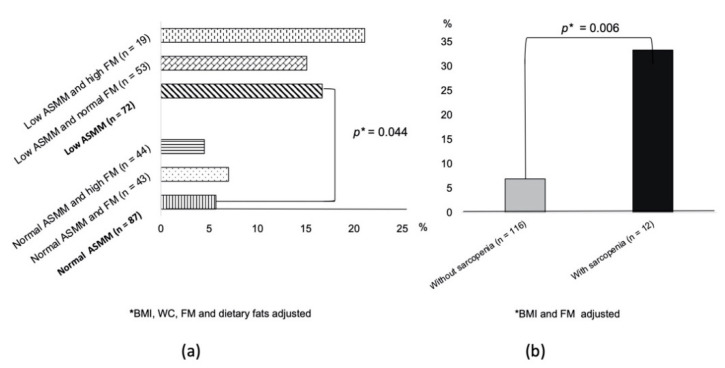
Incidence of impaired fasting glucose/type 2 diabetes according to appendicular skeletal muscle mass and fat mass categories (**a**) or the presence of sarcopenia (**b**).

Figure 3a shows that participants with a low ASMM had a higher IFG/T2D incidence than those in the normal ASMM group (17% vs. 6%), which was independent of FM, BMI, WC, and dietary fats (adjusted *p* = 0.044).

The OR of low ASMM for IFG/T2D onset was 3.81 (SE 0.55, *p* = 0.034, CI = 1.09–9.80). In the subgroup with data available on sarcopenia at the baseline (*n* = 128), elderly women with sarcopenia (Figure 3b) had a higher IFG/T2D incidence than those without sarcopenia (33% vs. 7%, adjusted for BMI and FM *p* = 0.005). In this subgroup of 128 women, the OR of sarcopenia for IFG/T2D onset was 6.75 (SE 0.71, *p* = 0.007, CI = 1.66–27.33). Supplemental Table S3 shows the changes in clinical parameters at the follow-up visit. More than 70% of the cohort was weight stable (73% and 83% in normal and low ASMM, respectively, *p* = 0.24). Figure 3a also shows the prevalence of IFG/T2D according to the different ASMM and FM categories. In particular, analysis using the chi-square test revealed that the final prevalence of IFG/T2D in women with low ASMM was not significantly different between those with high FM and those with normal FM. Furthermore, women with normal ASMM but high FM had the same prevalence of IFG/T2D as women with normal FM. All the clinical characteristics were not significantly different between the four groups according to ANOVA.

The area under the ROC curve for ASMM to predict the onset of IFG/T2D was 0.352 (SE = 0.066, *p* = 0.04, lower limit 0.224, higher limit 0.481) (figure not shown). An ASMM of 17.1 kg achieved a low sensitivity (10%) but a good specificity (80%) for predicting IFG/T2D. 

## 4. Discussion

Limited research has investigated the impact of muscle mass on the onset of T2D in elderly women; in particular, studies specifically designed to investigate the role of low ASMM in predicting T2D are lacking. In this retrospective study, we observed, for the first time, a significant difference in the incidence of IFG/T2D in a cohort of weight-stable elderly women that were categorized as normal and low ASMM at the baseline using BIA (Figure 3a). In particular, the probability of developing IFG/T2D over a mean follow-up of seven years was higher in women who had a low ASMM than in those with a normal ASMM, independent of the FM size (Figure 2a). Furthermore, women with sarcopenia had a higher probability of developing IFG/T2D than women without sarcopenia (Figure 2b). We found a baseline prevalence of sarcopenia of 9% for the whole study group. This finding is in line with a previous study reporting a prevalence of sarcopenia of 5–13% in 60- to 70-year-old individuals and 11–50% for people aged 80 or older [42]. The issue concerning the role of body composition in influencing the onset of T2D is controversial. Obesity is a key factor, although not the sole factor, in the increasing incidence of diabetes. According to several longitudinal studies, total LBM was positively associated with a high incidence of T2D, but no association remained significant after adjusting for FM [16,17]. In line with these two studies, Hong et al. reported a positive association between FM percentage and the incidence of T2D [22]. Other studies reported a high risk of T2D associated with a high FM size, also after adjusting for LBM [43,44]. The association between muscle indices and incident diabetes in well-functioning older adults living in a community is strongly influenced by their BMI category, especially for women [16]. In fact, in line with our findings, an 11-year follow-up of the Health ABC Study found a 40–60% decrease in the risk of developing T2D among normal-weight women with high muscle mass; however, a high muscle mass was associated with an increased risk of T2D in overweight and obese women [16].

In this study, measures of abdominal and thigh muscle were derived from single-slice CT scans and reported as areas, while total body fat, percentage FM, and total LBM were measured using dual-energy X-ray absorptiometry (DXA) [16]. According to our study, the authors assumed that a low skeletal muscle mass could play a role in the development of “normal-weight metabolic obesity” [16]. Thus, BMI acted as an effect modifier in the association between muscle mass and T2D risk and muscle mass played a crucial role as a preventive factor only in those without excess adiposity. However, it could be assumed that muscle mass is metabolically beneficial for all individuals but, in overweight/obese women, the harmful effects of excess adiposity overpowered the benefits of muscle [16]. However, a greater muscle lipid content was observed to be a characteristic feature of older adults with T2D [45,46]. This means that both intramyocellular lipids and intramuscular adipose tissue deposits can lead to the apparent increase in LBM, and thus explain the positive association between LBM and risk of diabetes in obese women [47].

In supporting the last concept, it was reported that the increased mortality risk for normal-weight patients, compared with overweight patients with T2D, appears to be mediated by their smaller relative muscle size [48]. Furthermore, the Rancho Bernardo Study highlighted that older individuals with sarcopenia have both a reduced LBM and FM compared with individuals without sarcopenia [49]. Considering all the previous studies and our results together, it emerged that if muscle mass was reduced, then normal weight, weight-stable, and sarcopenic subjects were also at risk of diabetes but their risk of diabetes did not depend on FM, as it did for the obese.

Of course, it is well known that CT and MRI are the gold standards for the quantitative estimation of adipose tissue distribution (area) [16]; however, these technologies are costly, have radiation issues, and cannot be routinely available. Therefore, we used a non-invasive and inexpensive method to study the body composition. 

In our study, we found a cumulative incidence of IFG/T2D of 11% over 7 years, and it was 8% among obese individuals. This finding is consistent with the 10-year incidence of T2D among Greek women in the ATTICA study [50], as well as the elderly participants in the Hoorn Study [41]. Despite having a high BMI, participants with normal ASMM had a lower incidence of T2D than those with lower BMI but low ASMM. This finding might have been due to the high prevalence of weight-stable individuals, which was more than 70% of the cohort. In fact, a twofold higher risk of T2D was demonstrated in participants whose body weight fluctuated compared with those with stable weight or moderate weight fluctuations over time [51]. Interestingly, overweight/obese but weight-stable elderly women had a lower risk of T2D than overweight/obese women with high body weight variability [52]. Indeed, weight cycling may cause an accumulation of visceral fat that would explain the increased metabolic risk independent of total adiposity [53].

This finding suggests the importance of reducing body weight fluctuations irrespective of the initial BMI in diabetes prevention.

The annual loss of muscle mass was reported to be 1–2% at the age of 50 years onwards [30,54]. By the age of 80, the average muscle loss is approximately 40% of the peak muscle mass that is usually attained at the age of 20 [55]. Older women have been reported to be more sedentary and less active than older men [56]. Since women have a longer life expectancy than men, this fact could imply that sarcopenia represents a greater health concern for women. Additionally, controversies exist about a more adequate way of considering LBM. Relative (percentage of body weight) versus absolute (kg) measures of LBM may have discordant relationships with the development of diabetes, as observed in the Baltimore Longitudinal Study of Aging [23], which included participants of both sexes that were much younger than those of our study. In fact, it was demonstrated that people in the highest quartile of percentage of LBM had the lowest probability of developing T2D; however, the opposite association was observed when the LBM was considered in absolute value. Our study focused on ASMM in elderly women, who are known to exhibit a higher prevalence of sarcopenia [55,57]. This condition could be linked with their low absolute muscle mass; they demonstrated 40% less upper body and 30% less lower body muscle mass than men [55]. Risk factors for the difference in muscle mass between gender are not well defined [58]. Gender differences in hormones are potential factors [58]. In general, older women have more disabilities than older men [58]. It is quite difficult to discriminate between the effects of aging and menopause, as both take place at the same time. With the menopausal transition, the decrease in estrogen seems to be related to an increase in oxidative stress and a decrease in insulin sensitivity, circulating IGF-1, DHEA, GH, and vitamin D, which are all related in some ways to the loss of muscle mass in women [59]. All these previous findings agree with our results demonstrating an important role of ASMM in predicting IGF/T2D in elderly women. As skeletal muscle is responsible for the majority of postprandial glucose disposal in the body, dysfunction of this part of the body might result in substantial whole-body metabolic disorders [60]. Physical inactivity, which is a common feature of aging, is associated with a decline in mitochondrial oxidative function in muscle [61]. This decline involves a reduced capacity to oxidize fatty acids, leading to insulin resistance (IR) and diabetes [62]. The REPOSI study [63], which is a collaborative study involving a network of hospitals in the European Union, demonstrated that women had a worse functional status than men, which could be related to the older age of women than men in that cohort, and it could also be the effect of a possible link between both depression and cognitive impairment and daily living activities [63].

Myostatin and adiponectin play important roles in skeletal muscle function by regulating insulin signaling and energy metabolism [64]. Myostatin is a member of the transforming growth factor beta superfamily and is an autocrine/paracrine inhibitor of skeletal muscle growth and development [65]. Myostatin is increased in sarcopenia [65] and plays a role in diabetic muscle atrophy [66,67]. Adiponectin is secreted mainly from the adipose tissue and has anti-atherogenic and anti-inflammatory actions [68,69]. Serum adiponectin levels increased with aging, and a high adiponectin concentration in the elderly is associated with a low muscle mass and strength [68]. It is not known whether it increases compensatively or due to low FM. Indeed patients with obesity have low serum adiponectin levels [69]. These previous studies could suggest that the skeletal muscle and adipose tissue may crosstalk to control glucose homeostasis. However, it is possible that changes in LBM represent both a risk factor and a consequence of impaired glucose tolerance.

Our study is of relevance considering that the relationship between hyperglycemia and cardiovascular disease (CVD) is a continuum. In the Rotterdam Study [70], among elderly participants with normal blood glucose and without diabetes, people with higher blood glucose levels also had higher arterial stiffness. Additionally, several studies demonstrated that elevated fasting glucose was associated with CVD and mortality in individuals without diabetes [2,71,72,73,74,75,76]. However, in our population, the increase in CV risk could have occurred due to other mechanisms and despite the good control of dyslipidemia using lipid-lowering agents (Table 2). Indeed the population studied was mostly hypertensive (more than 60%, Table 2). This leads to a progressive reduction in the glomerular filtration rate and loss of renal function over time. [77]. These individuals have lifelong exposure to cardiovascular risk [77].

Although the prevalence of sarcopenia increases with age, this condition is potentially reversible. In fact, previous studies showed that physical activity had a positive effect on muscle mass and function in elderly individuals [78,79]. Furthermore, protein intake was associated with a low risk of sarcopenia [60,80,81]. As previously reported [16], overweight/obese older individuals would likely benefit more from losing excess adiposity than building muscle mass, while normal-weight people would likely benefit more from maintenance or building muscle. Thus, increasing physical activity and protein intake could represent an important strategy for the treatment of individuals with a particular phenotype (i.e., sarcopenic, normal-weight individuals with glucose tolerance abnormalities). Thus, performing a BIA assessment would represent a new way to personalize the treatment, at least in the elderly. However, only intervention studies can confirm this hypothesis, which remains speculative.

This study has some limitations. Although evidence clarifying the role of estrogens on muscle mass in elderly women is lacking, it is possible to assume that the different roles of androgens and estrogens contribute to the sex disparity in skeletal muscle morphology and function [82]. Furthermore, adiponectin, which is inversely correlated with estradiol levels, seems to be the most important molecule released from fat cells potentially preventing diabetes in pre- and post-menopausal women [19,83].

However, investigating these effects was not among the objectives of the study and we did not assess their serum levels.

Additionally, we did not include men; therefore, we left some gaps in our understanding of the role of ASMM in diabetes onset. The REPOSI study [63] showed that men were more impaired than women in terms of cumulative illness burden with respect to severity and comorbidity and, in that study, fasting glucose level was higher in men than in women. Future research should include elderly men to improve our knowledge about the link between ASMM and T2D.

Furthermore, due to the lack of some HGS measurements, we assessed the presence of sarcopenia only in a subgroup of the cohort. However, our study encourages more research in the field of sarcopenia to definitively confirm its role in the development of glucose abnormalities. Finally, a greater muscle lipid content was observed to be a characteristic feature of older adults with T2D [45,46], and thus, based on our results, we cannot provide a definitive answer on the role of adipose tissue in predicting the development of T2D.

This study also has some points of strength. By dividing participants into body composition categories, we reduced confounding factors between fat and lean mass indices. It has been suggested that LBM may have a discordant relationship with the development of diabetes [23]. Consequently, we focused on the objective of considering a standardized measure of ASMM. Another strength of the current study includes the availability of a large cohort of women with comprehensive clinical follow-up; thus, undetected T2D cases are less likely to have occurred.

## 5. Conclusions

In this retrospective study, for the first time, we observed a greater probability of glucose abnormalities in elderly women with low ASMM or sarcopenia than women with normal ASMM. However, further studies are needed to address whether ASMM loss in older individuals, as well as in other age groups and in males, may lead to the development of type 2 diabetes.

## Figures and Tables

**Table 1:** Demographic, anthropometric, and clinical characteristics of the cohort.

	Participants (*n* = 159)
Age (years)	71 ± 5
Body weight (kg)	65.3 ± 11
BMI (kg/m^2^)	28 ± 4
Smokers (%)	8
Physical activity (%)	52
Hyperlipidemia (%)	42
Lipid-lowering agents (%)	24
Hypertension (%)	65
Antihypertensive agents (%)	57
Calcium/vitamin D supplementation (%)	31
BMI-defined obesity (%)	26
FM-defined obesity (%)	38
Low ASMM (%)	45
Sarcopenia * (%)	9.4
WC (cm)	92.8 ± 11
HC (cm)	103.7 ± 10
HGS * (kg)	19.6 ± 4
SBP (mmHg)	130 ± 16
DBP (mmHg)	78 ± 9
Bioimpedance analysis	
Rz (Ω)	554.8 ± 69
Xc (Ω)	54.0 ± 9
pA (°)	5.6 ± 0.8
FFM (kg)	22.1 ± 8.0
FM (%)	32.9 ± 6.2
ASMM (kg)	15.4 ± 2.1
Blood concentrations of (mg/dL)	
Glucose	89 ± 8
Creatinine	0.76 ± 0.2
Total cholesterol	215 ± 38
HDL cholesterol	64 ± 16
LDL cholesterol	131 ± 36
Triglycerides	106 ± 46

Data are given as mean ± SD or prevalence as appropriate. BMI, body mass index; WC, waist circumference; HC, hip circumference; HGS, handgrip strength; SBP, systolic blood pressure; DBP, diastolic blood pressure; HDL, high-density lipoprotein; LDL, low-density lipoprotein; Rz, resistance; Xc, reactance; pA, phase angle; FFM, fat-free mass; FM, fat mass; ASMM, appendicular skeletal muscle mass. * Only on 128 participants.

**Table 2:** Characteristics of the cohort that was classified according to appendicular skeletal muscle mass.

	ASMM		*p*
	Normal (*n* = 87)	Low (*n* = 72)	
ASMM (range, kg)	15–22.5	10.6–14.9	
Age (years)	70 ± 4	71 ± 6	0.36
Body weight (kg)	71.9 ± 10	57.3 ± 6	<0.001
BMI (kg/m^2^)	30.0 ± 4	26.0 ± 3	<0.001
Smokers (%)	7	9	0.77
Physical activity (%)	53	51	0.86
Hyperlipidemia (%)	36	49	0.10
Lipid-lowering agents (%)	20	29	0.19
Hypertension (%)	67	64	0.74
Antihypertensive agents (%)	60	54	0.52
Calcium and vitamin D supplementation (%)	26	36	0.30
BMI-defined obesity (%)	43	6	<0.001
FM-defined obesity (%)	51	22	<0.001
Sarcopenia * (%)	0	19	<0.001
WC (cm)	97.0 ± 12	87.6 ± 8	<0.001
HC (cm)	107.9 ± 9	98.7 ± 8	<0.001
HGS * (kg)	20.3 ± 3.9	18.8 ± 3.8	0.038
SBP (mmHg)	130 ± 17	129 ± 16	0.78
DBP (mmHg)	79 ± 8	77 ± 9	0.31
Bioimpedance analysis			
Rz (Ω)	519 ± 58	598 ± 57	<0.001
Xc (Ω)	53 ± 10	56 ± 9	0.06
pA (°)	5.8 ± 0.8	5.3 ± 0.7	<0.001
FM (kg)	25.6 ± 8.2	17.9 ± 4.0	<0.001
FM (%)	34.8 ± 6.3	30.7 ± 5.1	<0.001
Blood concentrations of (mg/dL)			
Glucose	88 ± 8	89 ± 7	0.54
Creatinine	0.78 ± 0.2	0.73 ± 0.1	0.06
Total cholesterol	217 ± 40	212 ± 37	0.50
HDL cholesterol	63 ± 16	65 ± 17	0.43
LDL cholesterol	133 ± 38	128 ± 35	0.38
Triglycerides	111 ± 52	100 ± 38	0.11

Data are mean ± SD or prevalence. Student’s unpaired *t*-test or chi-square test as appropriate. ASMM, appendicular skeletal muscle mass; BMI, body mass index; DBP, diastolic blood pressure; FFM, fat-free mass; FM, fat mass; HC, hip circumference; HDL, high-density lipoprotein; HGS, handgrip strength; LDL, low-density lipoprotein; pA, phase angle; Rz, resistance; SBP, systolic blood pressure; WC, waist circumference; Xc, reactance. * Only on 128 participants.

**Table 3:** Cox proportional hazards models for the risk of impaired fasting glucose/type 2 diabetes.

Panel A (*n* = 159)	Multivariate Analysis	
	HR (95% CI)	*p*
WC	0.98 (0.91–1.05)	0.58
BMI	1.08 (0.84–1.38)	0.53
High fat mass	0.94 (0.14–6.43)	0.95
Low ASMM	5.30 (1.34–21.04)	0.017
Fat intake (%)	1.00 (0.89–1.12)	0.92
Panel B (*n* = 128)	Multivariate Analysis	
	HR (95% CI)	*p*
BMI	0.95 (0.80–1.12)	0.58
High fat mass	1.70 (0.28–10.07)	0.55
Sarcopenia	4.72 (1.38–16.18)	0.013

ASMM, appendicular skeletal muscle mass; BMI, body mass index; CI, confidence interval; HR, hazard ratio; WC, waist circumference.

## Data Availability

The data presented in this study are available on request from the corresponding author.

## Supplementary Materials

The following are available online at https://www.mdpi.com/article/10.3390/nu13061909/s1. Table S1: Dietary intake assessment of the cohort classified according to appendicular skeletal muscle mass, Table S2: Changes in anthropometric parameters and blood glucose at follow-up according to the diagnosis of IFG/T2D, Table S3: Changes in clinical parameters at follow-up of the cohort classified according to appendicular skeletal muscle mass.
