# Supplementary material for: Sarcopenia and Appendicular Muscle Mass as Predictors of Impaired Fasting Glucose/Type 2 Diabetes in Elderly Women
Source: Nutrients. 2021 Jun 2;13(6):1909. doi: 10.3390/nu13061909 (PMC8227668; doi:10.3390/nu13061909)
Supplement: Supplementary file 1 [file nutrients-13-01909-s001.zip › nutrients-1226511-supplementary.pdf]

nutrients MDPI

Supplementary materials

Supplemental Table S1. Dietary intake assessment of the cohort classified according to appendicular skeletal muscle mass.

	ASMM		P[a]
	Normal	Low	
ASMM (range, kg)	15 - 22.5	10.6 - 14.9	
Calorie intake (kcal)	1535 ± 459	1677 ± 440	0.11
Carbohydrates[b] (%)	48.7 ± 8	46.4 ± 7	0.14
Proteins[b] (%)	18.8 ± 3	17.6 ± 3	0.06
Plant proteins[b] (%)	6.6 ± 1	6.1 ± 1	0.11
Animal proteins[b] (%)	12.1 ± 3	11.5 ± 4	0.39
Fats[b] (%)	31.3 ± 7	34.2 ± 5	0.019
Cholesterol (g)	204 ± 59	210 ± 87	0.71
Total fiber (g)	19.4 ± 7	20.9 ± 7	0.26
Alcohol (g)	5.7 ± 10	6.1 ± 9	0.82

Data are mean ± SD. [a] Student's unpaired t-test. [b] Adjusted for 1000/kcal
ASMM, appendicular skeletal muscle mass

Supplemental Table S2. Changes in anthropometric parameters and blood glucose at follow-up according to the diagnosis of IFG/T2D

	Glucose tolerance		P[a]
	Normal (n = 142)	IFG/T2D (n = 17)	
Age (years)	70 ± 5	72 ± 6	0.37
Follow-up time (months)	41 ± 23	39 ± 26	0.71
Weight (kg)	-0.53 ± 4	-0.73 ± 3	0.84
BMI (kg/m^2)	-0.15 ± 2	0.29 ± 1	0.22
BMI-defined obesity (%)	23	27	0.71
Blood glucose (mg/dL)	-1.4 ± 6	11.8 ± 9	<0.001

Data are mean ± SD or prevalence as appropriate. [a]Student's paired t-test or Student's unpaired t-test.
BMI, body mass index; IFG, impaired fasting glucose; T2D, type 2 diabetes.

Supplemental Table S3. Clinical characteristics at follow-up and changes of these parameters at follow-up of the cohort classified according to appendicular skeletal muscle mass.

	**ASMM**		P[a]
	**Normal**	**Low**	
ASMM (range, kg)	15 - 22.5	10.6 - 14.9	
Follow-up time (months)	43 ± 22	38 ± 24	0.18
Body weight (kg)	69.9 ± 11	57.5 ± 5	0.001
Body weight change (kg)	-1.3 ± 4	0.1 ± 3	0.06
BMI (kg/m^2)	29.8 ± 4	25.6 ± 2	<0.001
BMI change (kg/m^2)	-0.35 ± 2	0.13 ± 1	0.15
Weight stability (%)	73	83	0.24
Weight increase (>5%; %)	6	8	0.72
BMI-defined obesity (%)	42	6	<0.001
Blood glucose (mg/dL)	89 ± 7	90 ± 8	0.41
Blood glucose change (mg/dL)	-0.95 ± 8	1.53 ± 8	0.14

Data are mean ± SD or prevalence as appropriate. [a]Student's paired t-test.
ASMM, appendicular skeletal muscle mass; BMI, body mass index.
